# Indigenous Fungivorous Nematodes Affect the Biocontrol Efficacy of *Trichoderma harzianum* through Reducing the Hyphal Density

**DOI:** 10.4014/jmb.2102.02003

**Published:** 2021-03-30

**Authors:** Tae Gwan Kim, Guy R. Knudsen

**Affiliations:** 1 Department of Microbiology, Pusan National University, Pusan 46241, Republic of Korea; 2 Soil and Land Resources Division, Department of Plant, Soil, and Entomological Sciences, University of Idaho, Moscow, ID 83844, USA

**Keywords:** Biocontrol fungi, fungivorous nematodes, sclerotia, trophic interaction

## Abstract

Indigenous fungus-feeding nematodes may adversely affect the growth and activity of introduced biocontrol fungi. Alginate pellets of the biocontrol fungus *Trichoderma harzianum* ThzID1-M3 and sclerotia of the fungal plant pathogen *Sclerotinia sclerotiorum* were added to nonsterile soil at a soil water potential of -50 or -1,000 kPa. The biomass of ThzID1-M3, nematode populations, and extent of colonization of sclerotia by ThzID1-M3 were monitored over time. The presence of ThzID1-M3 increased the nematode population under both moisture regimes (*p* < 0.05), and fungivores comprised 69–75% of the nematode population. By day 5, the biomass of ThzID1-M3b and its colonization of sclerotia increased and were strongly correlated (*R*^2^ = 0.98), followed by a rapid reduction, under both regimes. At -50 kPa (the wetter of the two environments), fungal biomass and colonization by ThzID1-M3 were less, in the period from 5 to 20 days, while fungivores were more abundant. These results indicate that ThzID1-M3 stimulated the population growth of fungivorous nematodes, which in turn, reduced the biocontrol ability of the fungus to mycoparasitize sclerotia. However, colonization incidence reached 100% by day 5 and remained so for the experimental period under both regimes, although hyphal fragments disappeared by day 20. Our results suggest that indigenous fungivores are an important constraint for the biocontrol activity of introduced fungi, and sclerotia can provide spatial refuge for biocontrol fungi from the feeding activity of fungivorous nematodes.

## Introduction

*Sclerotinia sclerotiorum* is an important pathogenic fungus that has worldwide distribution and infects a wide variety of plants [1, 2]. Its sclerotia germinate either myceliogenically to produce vegetative hyphae, or carpogenically to produce airborne ascospores, which are considered as the primary inoculum for the pathogen for most plants [1]. *Sclerotinia* spp. generally spend about 90% of their life cycle as sclerotia in the soil [1], and sclerotia of *S. sclerotiorum* can survive for long periods of up to 7 or more years in field soils [3]. The most significant soil component affecting the survival of sclerotia might be biological [1]; numerous fungi, such as *Trichoderma* spp.(soilborne filamentous fungi), have frequently been found in decomposed sclerotia [1, 4]. *T. harzianum* has been extensively researched as a biocontrol agent against several soilborne fungal pathogens [5, 6]. *T. harzianum* is capable of suppressing carpogenic and myceliogenic germination from sclerotia of *S. sclerotiorum* [6, 7].

Soil nematodes (40-1,000 μm in length), as the most numerous soil-inhabiting animal, can be categorized into five trophic groups based on their feeding activities: bacterivores, fungivores, omnivores, predators, and plant parasites [8, 9]. Fungivorous nematodes have a stylet and feed on the hyphae of many species of soil fungi, both beneficial and pathogenic [10, 11]. Filamentous fungi can stimulate soil populations of fungivorous nematodes, such as *Aphelenchus* spp. and *Aphelenchoides* spp., which can then regulate fungal biomass and hyphal growth [12-14]. For example, Bae and Knudsen [14] reported that the addition of the nematode *Aphelenchoides* adversely affected the growth and activity of the introduced fungus *T. harzianum*, suggesting that fungivorous nematodes can be a significant constraint on the activity of biocontrol fungi in field soils. Recently, Kim and Knudsen [15] reported that fungal hyphae can support the population growth of indigenous nematodes when they are densely localized in soil, but not when homogeneously distributed.

Soil moisture is probably the most important abiotic factor for the growth and activity of nematodes and *Trichoderma* [16, 17], affecting nematodes more than fungi [18]. In general, the population growth of nematodes is best accomplished at high soil moisture levels, and their abundance is moisture dependent [19-21]. Ekschmitt and Griffiths [20] reported that the number of genera and abundance of fungivorous nematodes were highest in soil at around 30–40% water-holding capacity. In contrast, high levels of germination, growth, and activity of T. harzinaum are possible at a relatively low water potential [22-27]. Thus, at more negative soil water potentials, the population growth and feeding activity of nematodes on hyphae of *T. harzianum* may be reduced.

For mycoparasitism to occur in soil, hyphae of introduced biocontrol fungi must grow out and contact the target propagules (*e.g.*, sclerotia and hyphae) of pathogenic fungi to initiate mycoparasitism [5, 28, 29]. In the present study, we hypothesized that indigenous nematodes adversely affect the biocontrol efficacy of the introduced fungus *T. harzianum* ThzID1-M3. We tested whether the extent of colonization of sclerotia of *S. sclerotiorum* by ThzID1-M3 depends on the hyphal density of ThzID1-M3, whether fungivorous nematodes adversely affect the hyphal density of the introduced fungus, and whether soil moisture affects the biomass and biocontrol efficacy by regulating the activity of fungivorous nematodes. We also hypothesized that sclerotia provide spatial refuge to biocontrol fungi from predation by fungivores; the effect of fungivorous nematodes on colonization of sclerotia by biocontrol fungi may be quite small because their ability to access the biocontrol fungus would be much more difficult inside than outside sclerotia.

## Materials and Methods

### *T. harzianum* and *S. sclerotiorum*

*T. harzianum* ThzID1-M3 [30] is a transformant strain of *T. harzianum* ThzID1 [5], containing three exogenous genes encoding green fluorescent protein (GFP), ß-glucuronidase, and hygromycin B resistance. ThzID1-M3 was allowed to grow for 1 week on potato dextrose agar (PDA) plates at 25°C, then three 1-cm^2^ pieces from the sporulating culture were transferred to 1-L flasks containing 500ml potato dextrose broth (PDB) with streptomycin (25 μg/ml). They were incubated at room temperature on a rotary shaker (120 rpm) for 1 week, with 12 h of light per day. ThzID1-M3 cultures were strained through cheesecloth and rinsed thoroughly with sterile distilled water. Thirty-seven grams of hyphal biomass were briefly blended with 2 g of wheat bran and 100 ml of 1%aqueous sodium alginate solution. Drops of the mixture were added to 0.25 M aqueous CaCl_2_, forming precipitated pellets which were then air-dried at room temperature and stored at 4°C before use. The approximate amount of pellets used for the experiment was 17 mg.

*S. sclerotiorum* isolated from diseased lentil (*Lens culinaris* Medik.) [5] was grown on PDA plates for 1.5 months. Sclerotia formed on PDA are relatively regular in size. These sclerotia were harvested from the PDA plates, surface-sterilized for 1 min with a solution of 10% ethanol and 10% bleach in sterile distilled water, and washed twice with sterile distilled water. They were then air-dried at room temperature and stored at 4°C. The approximate amount of sclerotia used for the experiment was 13.1 mg.

### Soil and Experimental Conditions

The soil used was a Palouse silt loam (Pachic Ultic Haploxerolls) obtained from the University of Idaho Plant Science Farm, near Moscow. Soil analysis (University of Idaho Analytical Service Laboratory) showed that the soil contained 20% sand, 20% clay, and 60% silt by weight, and had a pH of 5.9. Collected soil was air-dried and sieved through a 2-mm mesh, prior to use. Soil was adjusted to a soil moisture content of -50 kPa or -1,000 kPa with sterile distilled water, prior to use. Three hundred grams of soil were added to individual containers, each being approximately 8 × 6.3 × 6.3 cm in size. A ThzID1-M3 pellet and a sclerotium were attached to a plastic toothpick (10 mm apart), using cyanoacrylate glue. The toothpicks were randomly assigned to containers and were placed vertically in the soil at a depth of approximately 5 cm (one toothpick per container). Lids were placed on the containers which were then sealed with parafilm to maintain relatively constant moisture levels. All experiments were conducted at 22±2°C, in a laboratory environment. There were four treatments: i) ThzID1-M3 and sclerotium at -50 kPa; ii) sclerotium only at -50 kPa; iii) ThzID1-M3 and sclerotium at -1,000 kPa; and iv) sclerotium only at -1,000 kPa. After 0, 5, 10, 20, and 40 days, approximately 10 g of soil was collected from around the toothpicks by using a steel corer with a diameter of 1.2 cm. There were 5 replicates for each treatment, at each sampling time.

### Nematode Population

Each soil sample was homogenized in 90 ml of 50 mM sodium phosphate buffer (pH 7.0) by agitation at 200 rpm for 1 h. To recover the nematodes, each soil suspension was placed on four layers of tissue paper in a Baermann funnel filled with distilled water. Extracts (about 2 ml) were collected every 2 days, over a 6-day period. A 1-mL aliquot was placed on a small Syracuse dish, and total nematodes were counted for each sample using a dissecting microscope at 32× magnification. Then, the nematodes were placed on slide glasses, killed by heat, and fixed by the addition of double-strength formaldehyde-acetic acid solution (100 ml 40% formaldehyde; 10 ml glacial acetic acid; and 390 ml distilled water). Nematode specimens were viewed with a Nikon Eclipse E1000 microscope (Nikon Corp., USA) from 400× to 1,000× magnification. Finally, the specimens were separated into two trophic groups, fungivores and others, by the presence of a stylet.

### Biomass of the Introduced Fungus in Soil

ThzID1-M3 biomass was measured based on GFP activity as previously described by Orr and Knudsen [31]. Briefly, 1 ml of a 1/100 dilution was passed through a non-fluorescent membrane filter (Millipore MF Black filter, 0.8 µm, 47 mm). Filters were viewed at 100× magnification under UV light for observing GFP activity on fungal hyphae with a Nikon Eclipse E1000 Epi-fluorescence microscope. On each captured image, radii and length of visible hyphae were quantified using ImagePro Plus imaging software (Media Cybernetics, USA). ThzID1-M3 biomass was calculated with the formula: fungal biomass (B) = π * r^2^ * L * d (r is the radius, L is the length, and d is the density of fungal hyphae).

### DNA Extraction

For DNA extraction, ThzID1-M3 was allowed to grow for 1 week on PDA at 25°C, then three 1-cm^2^ pieces from the culture were transferred to 1-L flasks containing 500 ml PDB. The flasks were incubated on a rotary shaker (120 rpm) for 4 days at room temperature. Mycelia were harvested by filtration through cheesecloth, rinsed with sterile distilled water, and ground with liquid nitrogen in a sterilized porcelain mortar. Total genomic DNA was extracted from the mycelia using the DNeasy Plant DNA Extraction Kit (Qiagen Inc., USA). DNA was eluted twice in 100 μl of AE buffer and both eluents were combined and stored at -20°C before use.

Sclerotia recovered from soil were washed three times with sterile distilled water. They were ground with liquid nitrogen in a sterilized porcelain mortar. DNA was extracted from ground sclerotia using the DNeasy Plant DNA Extraction Kit (Qiagen). DNA was eluted twice in 75 μl of AE buffer, and both eluents were combined and stored at -20°C before use.

### Quantitative PCR

The TMeGFP2 primer/Taqman probe set targeting the gfp gene was used for quantification of ThzID1-M3: TMeGFP2-foward (5'-GCTGCCCGACAACCACTAC-3'), TMeGFP2-reverse (5'-CGTCCATGCCGAGAGTGATC-3'), and TMeGFP2-probe (5'-FAM-CGGCGGCGGTCACGAACTCCA-TAMRA-3') [32]. For a quantitative PCR (qPCR) assay, a 10-fold dilution series of ThzID1-M3 genomic DNA was constructed, ranging from 0.17 pg to 17 ng. qPCR reactions were carried out in a real-time PCR plate (VWR International, West Chester, PA, USA) in a total volume of 25 μl using the Bio-Rad iCycler IQ System (Bio-Rad, Hercules, CA, USA). The reaction mixture contained 2.5 μl of 10× PCR buffer (Invitrogen, Carlsbad, CA, USA), 0.5 μl of 10 mM dNTPs mixture (Invitrogen), 2 μl of 50 mM MgCl_2_ (Invitrogen), 0.1 μl (0.5 unit) of Platinum Taq DNA Polymerase (Invitrogen), 0.75 μl of TMeGFP2F (10 μM), 0.25 μl of TMeGFP2R (10 μM), 0.25 μl of TMeGFP2P probe (5 μM), and 5 μl of DNA template. The cycling condition was an initial denaturation at 95°C for 2 min, followed by 40 cycles at 95°C for 20 s, and 60.5°C for 1 min.

### Microbial Population

A subsample (1 ml) of each soil suspension was serially diluted in 9 ml of the phosphate buffer. Aliquots (0.1 ml) were spread on tryptic soy agar plates with cycloheximide (50 μg/ml) for counting recoverable bacteria on *Trichoderma*-semi-selective medium (TSM) agar plates [33] for *Trichoderma* spp., and on TSM agar plates with hygromycin B (50 µg/ml) for ThzID1-M3. After incubation for 5 days at 23°C, the CFUs were counted. Colonies of *Trichoderma* spp. and ThzID1-M3 were determined by color and morphology and were counted on each of the selective media.

### Data Analysis

This experiment was conducted as a completely randomized block design (moisture levels and presence of ThzID1-M3 pellet treated as factors, and sampling times as blocks). All analyses were performed using SAS package 9.0 (SAS Institute Inc., USA). Recoverable numbers of bacteria, *Trichoderma* spp., and ThzID1-M3 were standardized by gravimetric soil moisture and log-transformed prior to analysis. Analysis of variance using PROC GLM was performed for analyzing microbial populations and ThzID1-M3 biomass in soil, and for ThzID1-M3 DNA estimates in sclerotia, with significant differences at *p* < 0.05.

## Results

### Total and Fungivorous Nematode Populations

**Fig. 1:**
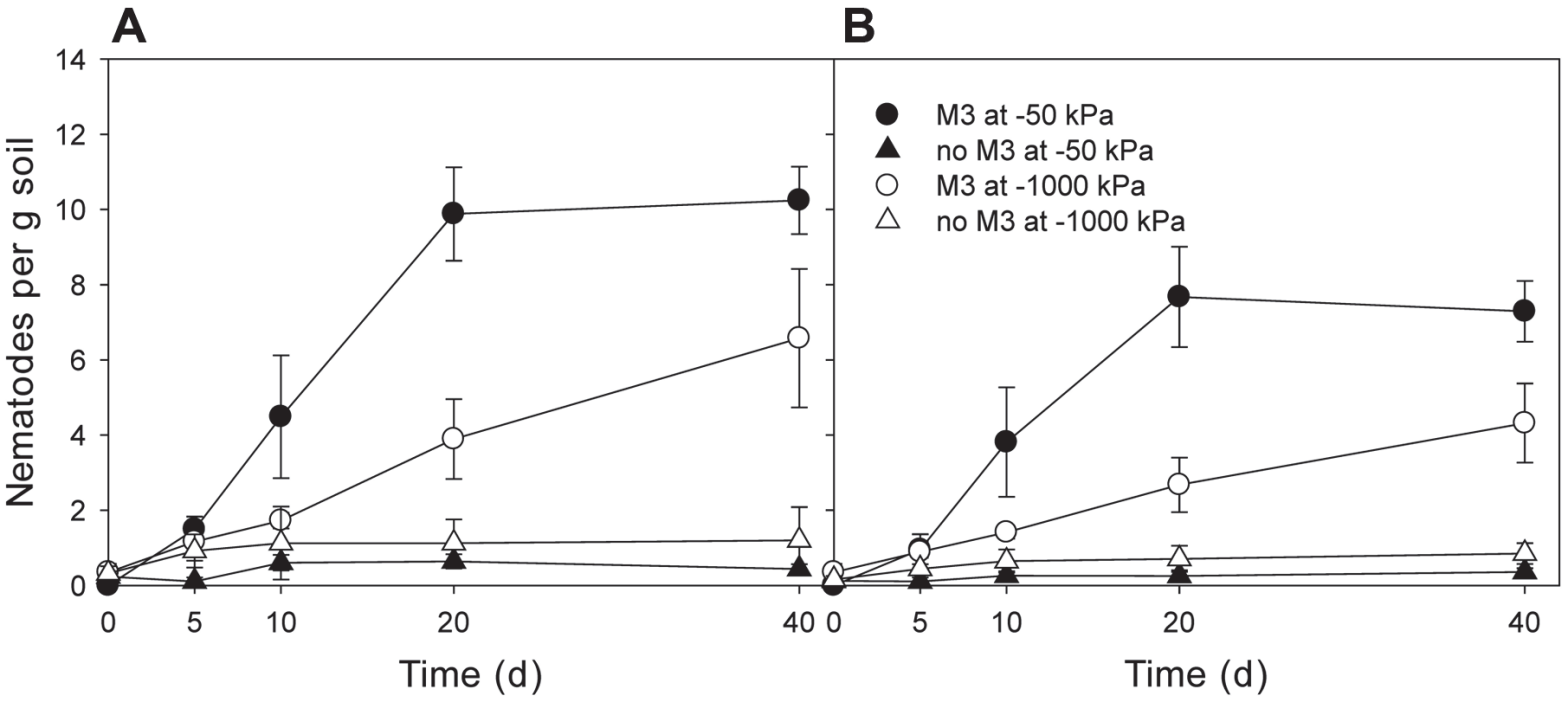
Temporal population changes of total (A) and fungivorous (B) nematodes in soil. Error bars represent 1 standard error of the mean.

The addition of ThzID1-M3 increased both total and fungivorous nematode populations (*p* < 0.05) (Fig. 1). Neither type of nematode would grow in the absence of ThzID1-M3. Soil moisture affected the growth of both nematode populations (*p* < 0.05). There was a significant interaction between ThzID1-M3 and soil moisture for total and fungivorous nematodes (*p* < 0.05). In the presence of ThzID1-M3, both populations were greater at -50 kPa than at -1,000 kPa (*p* < 0.05). Fungivores comprised about 75% and 69% of the total nematode population at -50 kPa and -1,000 kPa, respectively. At -50 kPa, both populations peaked on day 20 and did not change, while at -1,000 kPa, they gradually increased until day 40.

### ThzID1-M3 Biomass in Soil

**Fig. 2:**
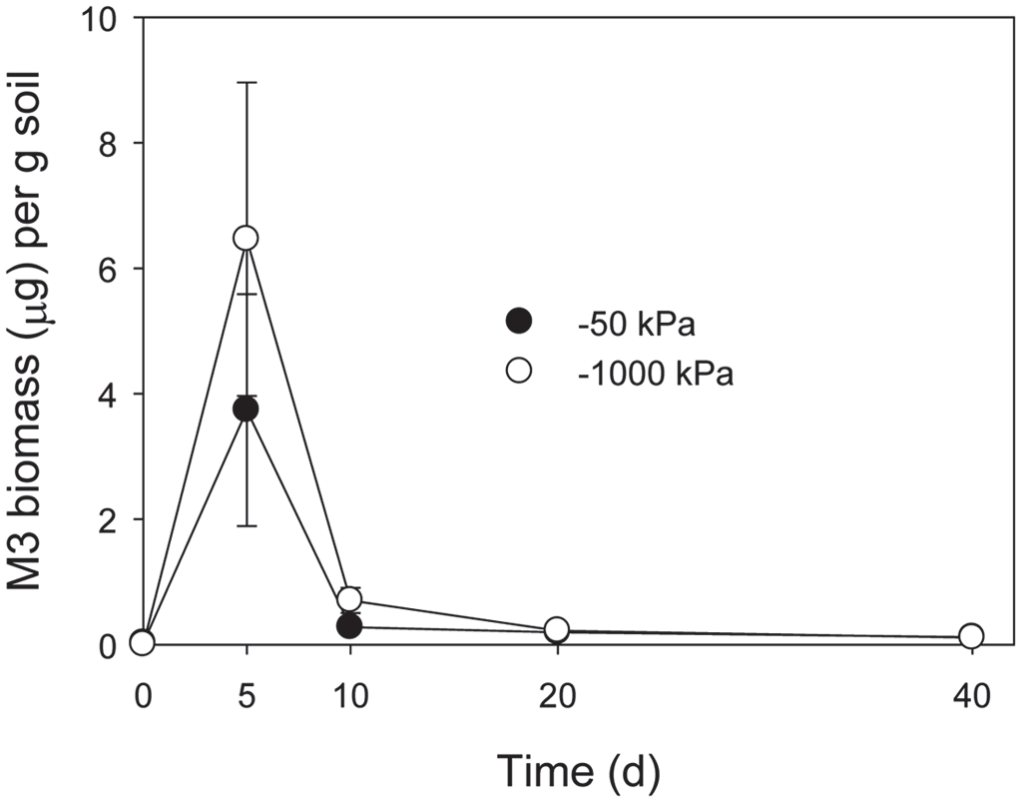
Temporal variation of *T. harzianum* ThzID1-M3 in soil. Error bars represent 1 standard error of the mean.

ThzID1-M3 biomass peaked on day 5, then sharply decreased by day 10, at both soil moisture levels (Fig. 2), and by day 20, ThzID1-M3 hyphae were undetectable, although dozens of resting structures were observed. ThzID1-M3 biomass appeared to be greater at -1,000 kPa than at -50 kPa in the period from 5 to 20 days (no statistical significance shown, *p* > 0.05).

### Colonization of Sclerotia by ThzID1-M3

**Fig. 3:**
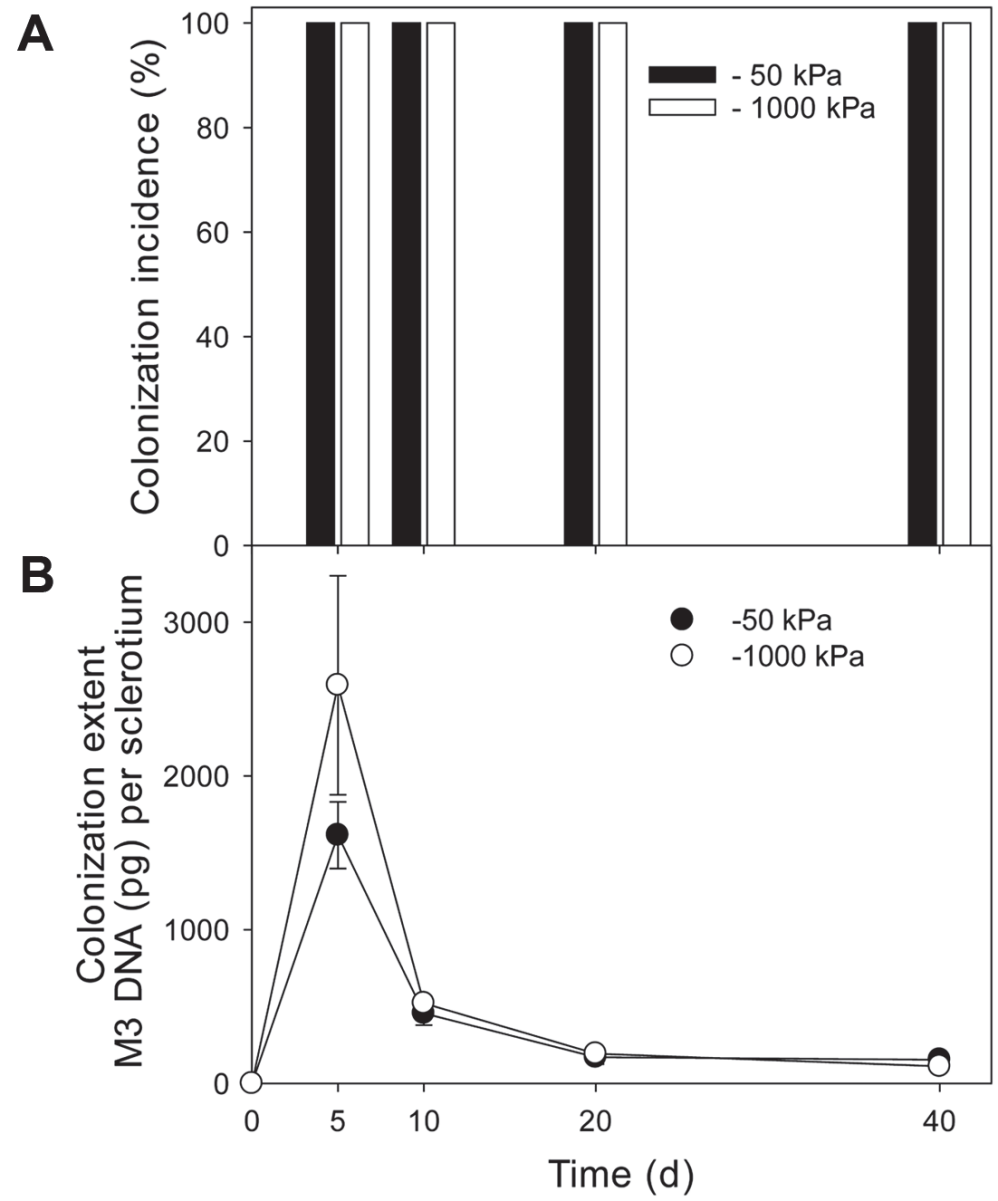
Colonization of sclerotia by *T. harzianum* ThzID1-M3. Colonization incidence (**A**) and extent (**B**) by ThzID1-M3. Extent was determined on 5 sclerotia per treatment at 0, 5, 10, 20, and 40 days using quantitative PCR. Error bars represent ±1 standard error of the mean.

Colonization of sclerotia by ThzID1-M3 was quantified using qPCR. The standard curves obtained from the qPCR assay with the TMeGFP2 set generated a linear fit, with a slope of -3.4 to -3.6 and *R*^2^ > 0.99. They showed more than 90% PCR efficiency over at least 7 orders of magnitude, ranging from 170 fg to 170 ng of ThzID1-M3 genomic DNA. ThzID1-M3 colonized all sclerotia by day 5 (colonization incidence, 100%), and the colonization incidence remained until the end of the incubation period, at both moisture levels (Fig. 3A). The extent of colonization by ThzID1-M3 peaked on day 5, followed by a reduction (Fig. 3B). Colonization extent remained at a certain level over a 20- to 40-day period, ranging from 111 to 193 pg of ThzID1-M3 DNA. Estimates of ThzID1-M3 DNA in sclerotia were greater at -1,000 kPa than at -50 kPa, in the period from 5 to 20 days (no statistical significance shown, *p* > 0.05). None of the sclerotia germinated, myceliogenically or carpogenically, throughout the experimental period.

There was a strong correlation between the mean measurements of *T. harzianum* ThzID1-M3 biomass in soil and the mean estimates of ThzID1-M3 DNA in sclerotia (*p* < 0.05, *R^2^* = 0.98) (data not shown). The result indicates that the extent of colonization of sclerotia by ThzID1-M3 depends on the local hyphal density of ThzID1-M3.

### Microbial Populations

**Fig. 4:**
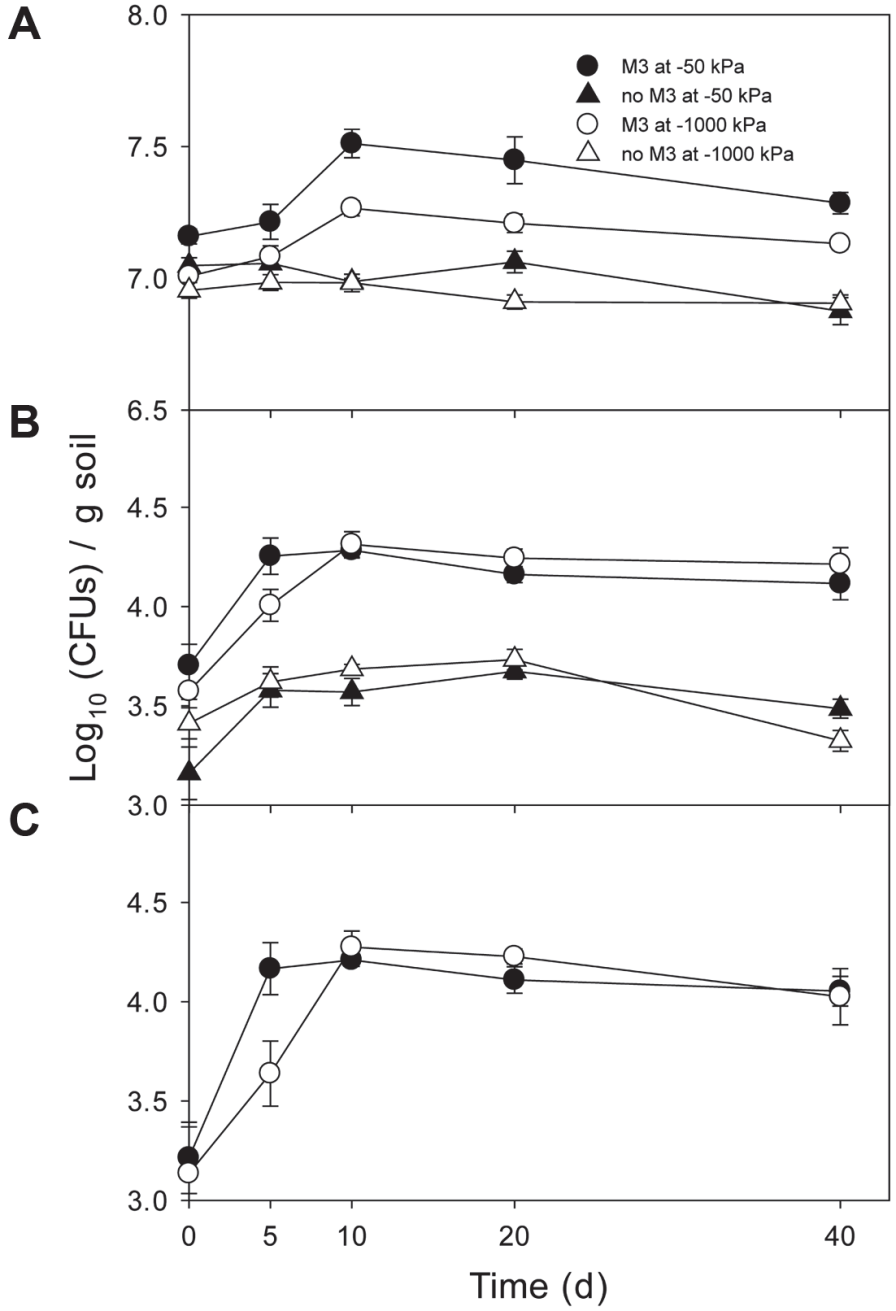
Temporal population changes of bacteria (A), *Trichoderma* spp. (B), and *T. harzianum* ThzID1-M3 (C) in soil. Error bars represent ±1 standard error of the mean.

The presence of ThzID1-M3 increased the recoverable number of total bacteria (*p* < 0.05) (Fig. 4A). With ThzID1-M3, at both soil moisture levels, bacterial populations peaked on day 10 and then decreased slightly over time; without ThzID1-M3, the bacterial population just slightly decreased over time. There was a significant interaction between ThzID1-M3 and soil moisture, affecting the bacterial population (*p* < 0.05). Bacterial populations were greater in the presence of ThzID1-M3 at -50 kPa than at -1,000 kPa (*p* < 0.05). When trends of bacterial populations over time were compared with those of total nematode populations, a correlation was seen (*p* < 0.05). ThzID1-M3 increased the populations of *Trichoderma* spp. at both moisture levels (*p* < 0.05) (Fig. 4B). The presence of ThzID1-M3 increased the number of recoverable *Trichoderma* spp. propagules (Figs. 4B, 4C). Soil moisture did not affect the populations of *Trichoderma* spp. and ThzID1-M3 (*p* > 0.05). Populations of *Trichoderma* spp. and ThzID1-M3 peaked on day 10 and did not undergo further changes with time.

## Discussion

Bae and Knudsen [14] reported that the addition of a fungivorous nematode can significantly reduce the radial growth and recoverable number of ThzID1-M3 introduced into the soil, and suggested the importance of fungivorous nematodes in regulating the biocontrol activity of an introduced fungus. The addition of ThzID1-M3 strongly stimulated the growth of nematode populations, in both soil moisture regimes, while there was no growth of nematode populations without the fungus (Fig. 1). Fungal hyphae can support the population growth of soil nematodes when they are densely localized [15]. We confirmed that fungivores comprise 69–75% of the total nematode population (Fig. 1). A previous study showed that ThzID1-M3 biomass extensively proliferated within 5 days of the addition of pellets, followed by a rapid reduction (Fig. 2), and a significant hyphal density of ThzID1-M3 was shown over a 5- to 40- day period when nematodes were eliminated from field soil [15]. Moreover, fungivorous nematodes such as *Aphelenchoides* and *Aphelenchus* are generally attracted to soil filamentous fungi [10]. Thus, we presumed that fungivorous nematodes selectively fed on hyphae of the fungus, originating from the alginate pellet. This may represent the way in which fungivorous nematodes regulate fungal biomass in natural soil environments. Our results suggest that fungivorous nematodes, one of the biotic constituents of soil, can have a profound effect on the survival and growth of biocontrol fungi in soil.

A sclerotium of *S. sclerotiorum* is a hyphal aggregate with an outer black rind, several cells thick and containing melanin pigments [34]. The melanized rind plays an important role in protection from adverse conditions [34, 35] and invasion by microorganisms [35, 36]. Hyphae of *Trichoderma* make contact with sclerotia of plant pathogenic fungi, forming a dense mycelium on the surface of the sclerotia to penetrate the rind and mycoparasitize the inside [37, 38]. The biomass of ThzID1-M3 in soil and the extent of colonization of sclerotia by ThzID1-M3 peaked on day 5, then significantly decreased by day 10. ThzID1-M3 hyphae that formed dense mycelia at the soil-sclerotia interface, as part of the process of mycoparasitization, were grazed by fungivorous nematodes. Thus, the extent of surface colonization decreased along with the reduction of ThzID1-M3 biomass in soil. Accordingly, there was a strong correlation between the ThzID1-M3 biomass and the extent of colonization of sclerotia by ThzID1-M3 (*p* < 0.05, *R^2^* = 0.98). ThzID1-M3 propagules that were indicative of a recoverable population, such as chlamydospores and conidia, did not correlate with the extent of colonization (Figs. 2–4). These results indicate that the extent of colonization depended on the hyphal density of ThzID1-M3 around the sclerotia. Overall, the mean hyphal biomass of the biocontrol fungus was greater, and thus the biocontrol efficacy was higher, at -1,000 kPa than at -50 kPa. This is in line with previous observations reporting that a greater hyphal density of *T. harzianum* ThzID1 resulted in a higher incidence of colonization of sclerotia by ThzID1, in *S. sclerotiorum* [5, 28]. By day 5, ThzID1-M3 colonized all sclerotia located 10 mm away from the alginate pellets during the initial proliferation of the fungus and this colonization was resilient over time (Fig. 3). Previously, when sclerotia and alginate pellets were randomly distributed in nonsterile soil, less than 30% of sclerotia were colonized by ThzID1-M3 [14, 39], while all sclerotia in the vicinity of alginate pellets were colonized by the fungus in the present study. In order to mycoparasitize the sclerotia, hyphae of the fungal agent must grow through the three-dimensional soil space and make contact with target sclerotia. Therefore, the extent of mycoparasitism depends on the spatial arrangement between sclerotia and fungal agents.

Soil moisture is probably the most important abiotic factor governing the growth and activity of soil organisms, although the limit of tolerance to decreasing soil moisture varies with taxa [16-18]. Soil nematodes find the best conditions for their population growth at relatively high moisture levels and are more sensitive to the drying of soil than are fungi [18-20, 40, 41]. The growth and activities of *T. harzianum* are generally reduced with decreased water potential, but their optimum range has been shown to be relatively wide, and they can operate at lower water potentials than can nematodes [22-27]. Total and fungivorous nematode populations were significantly greater in the wetter soil, with ThzID1-M3 (*p* < 0.05) (Fig. 1). Both populations peaked on day 20 and then did not change, at -50 kPa, while gradually increasing over the course of the experiment at -1,000 kPa. Although the population of fungivorous nematodes was significantly lower in the drier soil (*p* < 0.05), the biomass of ThzID1-M3 did not differ between moisture regimes (*p* >0.05). There were a considerable number of fungivores in both moisture regimes, capable of consuming the hyphal biomass of ThzID1-M3. A single fungivorous nematode can consume a significant amount of fungal biomass; for example, the consumption rate of fungal cytoplasm by the fungivore *Aphelenchus avenae* was calculated to be 0.12 μg per day [13].

ThzID1-M3 significantly stimulated the population growth of indigenous fungivorous nematodes, around the pellet and sclerotium, at both soil moisture levels (*p* < 0.05). However, ThzID1-M3 colonized all sclerotia by day 5 and maintained its colonization over the course of the experiment (Fig. 3). Although ThzID1-M3 hyphal fragments disappeared by day 20, and dozens of conidia and chlamydospores were seen over the period from day 20 to day 40, the extent of colonization was maintained at a constant level. The number of fungivorous nematodes was significantly greater at -50 kPa than at -1,000 kPa (*p* < 0.05), but this did not enhance the extent of colonization over the period in the wetter soil. The mean extent of colonization remained at 111–193 pg of genomic DNA, in the period from day 20 to day 40, equivalent to 5–8.8 μg of hyphal biomass. One milligram of dry ThzID1-M3 mycelia is equivalent to 21.40 ng of genomic DNA, by extrapolation of the linear relationship between weight measurements of dry mycelia and their DNA estimates, by qPCR assay (data not shown) (*p* < 0.05, *R^2^* = 0.97). Fungivorous nematodes are capable of decreasing hyphal density and growth of the biocontrol fungus, thus reducing the extent of mycoparasitism outside of sclerotia, whereas they have little or no effect on the extent of colonization inside sclerotia. Therefore, sclerotia provided a spatial refuge for the biocontrol fungus away from the feeding activity of fungivorous nematodes. Moreover, our results indicate that careful consideration of the formulation and/or delivery system of the biocontrol fungi is required for their successful application in field soils due to biotic factors. For example, low-density hyphal growth of biocontrol fungi may help avoid the significant growth of indigenous predators.

The added ThzID1-M3 stimulated the population growth of total culturable bacteria and generic *Trichoderma* (Fig. 4). In the presence of ThzID1-M3, the bacterial population was significantly greater at -50 kPa than at -1000 kPa, similarly to the nematode population. We observed a correlation between the total and fungivorous nematode populations and the bacterial populations over time (*p* < 0.05). The bacterial population increased along with fungivorous nematodes, which are capable of consuming ThzID1-M3 hyphae, because soil bacteria can rapidly decompose dead fungal hyphae [36]. Ingham *et al*. [13] suggested possible mechanisms for the stimulation of bacterial growth; that nematodes might transport bacterial cells either internally or externally on their cuticle to a substrate-rich microhabitat, where their excretory products would provide substrates or inorganic nutrients for bacterial growth. The majority of recoverable propagules of *Trichoderma* spp. resulted from the introduced ThzID1-M3 (Figs. 4B, 4C). As the ThzID1-M3 biomass increased, the *Trichoderma* and ThzID1-M3 recoverable populations increased, but did not then decrease with the subsequent reduction of ThzID1-M3 biomass, over time. These results indicate that recoverable numbers of *Trichoderma* propagules are not aligned with the biomass and biocontrol efficacy of *Trichoderma*.

The feeding activity of indigenous nematodes on hyphae of *T. harzianum* ThzID1-M3 adversely affected the biocontrol efficacy of the fungus because sclerotial colonization by ThzID1-M3 was dependent on its hyphal density in the soil. However, once sclerotia were initially colonized, an increased population of fungivorous nematodes did not affect the incidence of colonization of sclerotia by ThzID1-M3 or the extent of colonization, after ThzID1-M3 hyphae disappeared from the soil. These results suggest that fungivorous nematodes are a significant constraint on the hyphal proliferation and efficacy of biocontrol fungi while also indicating that sclerotia may provide spatial refuge to mycoparasitic fungi from the feeding activity of such nematodes.
